# A Comparison of Rehospitalization Risks on Diabetic and Non-Diabetic Patients after Recovery from Acute Coronary Syndrome

**DOI:** 10.3390/healthcare10061003

**Published:** 2022-05-28

**Authors:** Ho-Pang Yang, Shao-Jen Weng, Zih-Ping Ho, Yeong-Yuh Xu, Shih-Chia Liu, Yao-Te Tsai

**Affiliations:** 1 Department of Cardiology, Show Chwan Memorial Hospital, Changhua 50008, Taiwan; yanghopang@yahoo.com.tw; 2 Department of Industrial Engineering and Enterprise Information, Tunghai University, Taichung 40704, Taiwan; sjweng@thu.edu.tw; 3 Healthcare Systems Consortium, Tunghai University, Taichung 40704, Taiwan; 4 Department of Medical Research, Show Chwan Memorial Hospital, Changhua 50008, Taiwan; zihpingho@gmail.com; 5 Department of Computer Science and Information Engineering, Hungkuang University, Taichung 43302, Taiwan; yyxu@sunrise.hk.edu.tw; 6 Department of International Business, Feng Chia University, Taichung 40723, Taiwan

**Keywords:** LDL-C, acute coronary syndrome, diabetes

## Abstract

Background: The purpose of this study is to investigate whether the risks of rehospitalization caused by acute coronary syndrome (ACS) or stroke would have significant differences between diabetic and non-diabetic patients from ACS. Methods: This was a retrospective study of 364 inpatients with ACS from 2017 to 2019. Logistic regression models included gender, age group, and the principal diagnosis of hospitalization as controlling variables which were used to analyze the dataset. Results: About 10% of patients are hospitalized after recovery. Moreover, regardless of suffering from diabetes, the risk of rehospitalization does not appear to show a significant difference. In comparison with non-diabetic patients, the odds ratio of rehospitalization of diabetic patients was 0.94 (95% CI: 0.46-1.93, *p*-value = 0.8639) after controlling for the effects of gender, age group, and the principal diagnosis of hospitalization. Conclusions: Diabetic patients seem to perform well in controlling LDL-C (low-density lipoprotein cholesterol) after ACS recoveries.

## 1. Introduction

Along with the advancement in the economy and healthcare in society, the population and quality of life in Taiwan are experiencing intense change. However, overwork incidents have been appearing frequently in recent years. There are cases of sudden cardiac death of healthcare workers, high-tech personnel, security guards, police, and mailmen seemingly caused by work. A lack of sleep caused by working overtime, working shifts, and long commuting time [1,2,3] coupled with unhealthy living habits [4,5] is correlated to cardiovascular diseases. In addition, physiological changes caused by work pressure are factors in worsening cardiovascular diseases [6].

According to the statistics of the Ministry of Health and Welfare in Taiwan, cancer is continuously ranked among the top of the 10 leading causes of death, but cardiovascular diseases (including heart diseases and hypertensive diseases) are continuously ranked second or third in the 10 leading causes of death [7]. The World Health Organization also reports that an unhealthy diet, lack of physical activity, smoking, and drinking are major behavioral risk factors for cardiovascular diseases; meanwhile, cardiovascular diseases are the number one cause of death and have huge effects on low-income nations [8].

Disease burden is a major problem that people cannot ignore. The estimated cost directly derived from cardiovascular disease and the discussion of economic benefits, such as the economic cost resulting from smoking-induced cardiovascular disease [9], the evaluation of expenses for cardiovascular disease [10], and the cost-effectiveness and economic burden of cardiovascular disease treatment [11], should be considered.

Diabetes is a common chronic disease in the elderly population. In Taiwan, among the 10 leading causes of death, cerebrovascular disease, cardiovascular disease, and kidney disease, are closely related to poor diabetes control. It has been shown that diabetes has a huge impact on citizens [12]. Nevertheless, the primary symptoms of hypertension and diabetes are not obvious. Some patients are aware of suffering from diabetes or hypertension after the occurrence of acute cardiovascular disease or other complications [13,14], such as acute coronary syndrome (ACS). It is easily overlooked and can cause a high incidence of disease burden [9,10,11].

Hypercholesterolemia is regarded as a risk factor for ACS and is associated with higher mortality [15]. In general, the current prognostic treatment of ACS patients is mostly aimed at reducing the patient’s low-density lipoprotein cholesterol (LDL-C). Using statins could reduce the mortality rate and the recurrence of ACS [16,17], while for diabetics and high-risk groups, moderate-intensity statins (including Atorvastatin, Simvastatin, Lovastatin, Fluvastatin, Pitavastatin, Rosuvastatin, and Pravastatin) are usually used in the prognosis to prevent the risk of rehospitalization.

The international clinical research in IMPROVE-IT (IMProved Re-duction of Outcomes: Vytorin Efficacy International Trial) [18] addressed two issues of concern regarding: (1) the effectiveness of applying non-statin drugs to reduce LDL-C and (2) the benefit of continuously reducing LDL-C when it was at the low standard. The results revealed that treatment with the combination of ezetimibe and statins was effective in reducing patients’ LDL-C and reducing cardiovascular incidents. Nonetheless, statins also had side effects, including increasing AST/ALT and rhabdomyolysis; some data even considered that statins would result in diabetes and a decrease in cognitive function. The study in question pointed out the greater benefit of using ezetimibe for patients with diabetes, possibly because of the higher baseline risk; additionally, the LDL-C and high-sensitivity C-reactive protein of patients with diabetes were reduced more noticeably. It is mostly considered that the analysis could not reveal the unhelpfulness of ezetimibe in non-diabetic patients. However, some studies suggest that regular doses are sufficient in most cases [19,20], while others suggest that high-dose statin medication increases the risk of de novo diabetes [21] and the risk of elevated liver enzymes, rhabdomyolysis, and cancer [22].

Therefore, in order to assist ACS patients in coping with future major cardiovascular disease treatment, this study aims to examine the rehospitalization risk due to ACS or stroke for diabetic and non-diabetic patients after recovering from ACS.

## 2. Materials and Methods

**Figure 1:**
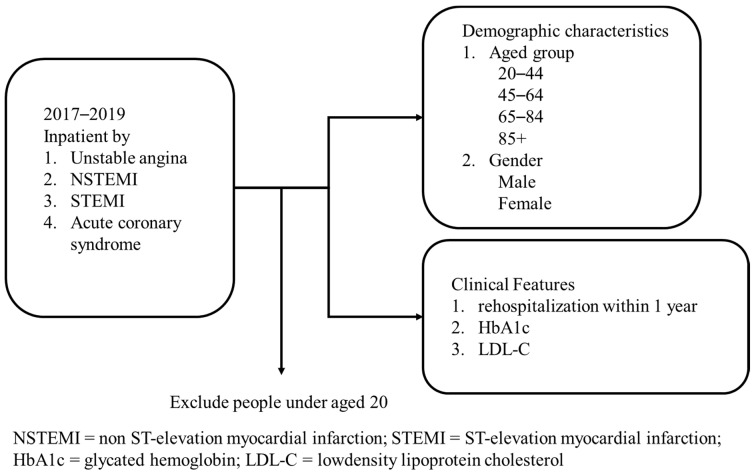
The process of the study population in the hospital.

The study was a retrospective analysis of the recovery effect from acute coronary syndrome (ACS). Inpatients with ACS at a regional hospital in central Taiwan from January 2017 to December 2019 were used as the data source for this study. These inpatients will return as outpatients once a month for three months after discharge, and they will return to the hospital for examination and tracking every 3–6 months. Patients’ demographic characteristics (including the age groups, 20–44, 45–64, 65–84, and 85+, and gender) and principal diagnosis (including unstable angina, non ST-elevation myocardial infarction (NSTEMI), ST-elevation myocardial infarction (STEMI), and acute coronary syndrome) were used as control variables in the analysis. In addition, the trends of LDL-C on non-diabetic and diabetic patients, and the risk of rehospitalization due to ACS or stroke within a year of discharge, were also obtained by this study. The case inclusion criteria are shown in Figure 1.

In terms of the classification of principal diagnosis codes, ACS of ICD-10 contains Unstable angina (I200), NSTEMI (I214), STEMI (I2101, I2102, I2109, I2111, I2119, I2121, I2129, I213), and ACS (I249). Furthermore, glycated hemoglobin (HbA1c) and low-density lipoprotein test data are collected for this study. The classification of diabetes is referred to as the patient’s HbA1c being higher than or equal to 6.5.

The chi-square test is used in this study for testing the difference in demographic characteristics, and the nonparametric Wilcoxon two-sample test is used to test the difference in low-density lipoprotein cholesterol (LDL-C) among patients with or without diabetes. Furthermore, the cases are observed involving rehospitalization caused by ACS or stroke within a year after being discharged, as the response variable. A multivariate logistic regression model was applied to examine the risk of rehospitalization within one year after ACS recoveries.

## 3. Results

The characteristics of these inpatients are shown in Table 1. Among the 364 patients, males appear the most, at 62.36%. Four age groups are divided in this study, namely 20–44, 45–64, 65–84, and 85 and over; and their percentages are 7.14%, 34.89%, 47.25%, and 10.71%, respectively. Most cases are older adults aged above 65, about 67.96% of all cases. Regarding the entire research case, the average age is 67. In terms of principal diagnosis, the percentages for diagnoses of unstable angina, NSTEMI, STEMI, and ACS stand at 9.34%, 39.01%, 48.08%, and 3.57%, respectively. Most ACS diagnoses are NSTEMI and STEMI.

Table 2 shows the summarized statistics (mean, std, Q1, median, and Q3) of LDL-C by non-diabetics (HbA1c < 6.5) and diabetics (HbA1c ≥ 6.5). Diabetic patients have a higher LDL-C than that of non-diabetic patients in all statistics. However, the difference between these two groups does not reach significant statistical differences in terms of LDL-C (*p*-value = 0.0883).

Table 3 presents the results of the logistic regression models. The rehospitalization risks caused by ACS or stroke within one year between diabetic and non-diabetic patients did not amount to a significant difference after controlling for the effects of gender, age, and principal diagnosis. Odds ratios of rehospitalization in univariate analysis and multivariate analysis were 1.08 (95% CI: 0.55–2.14, *p*-value = 0.8246) and 0.94 (95% CI: 0.46–1.93, *p*-value = 0.8639), respectively. Moreover, there do not appear to be significant differences—in terms of the effects of gender, age group, and the clinical hospitalization diagnosis—on the risk of rehospitalization caused by ACS or stroke within one year.

## 4. Discussion

Chronic diseases are major factors in elderly death, including malignant tumors, heart disease, cerebrovascular disease, diabetes, and hypertension. Along with reducing metabolic activity, humans could easily suffer from chronic diseases and complications and increase the death risk. Recently, the Taiwan Health Promotion Administration positively promoted the prevention of metabolic syndromes by testing for abdominal obesity, hypertension, hypertriglyceridemia, and hyperglycemia to cope with the threat of relevant diseases as early as possible. Nevertheless, the primary symptoms of hypertension and diabetes are not obvious and patients often only notice the symptoms of diabetes or hypertension after the occurrence of cardiovascular diseases or other complications [13,14]. Regarding patients with cardiovascular disease, drug control, an increase in physical activity, and reduction in unhealthy habits such as smoking could effectively improve the physical conditions to further reduce the risk of death [23,24,25]. Research also indicated that an increase in physical activity could effectively promote patients’ quality of life and self-rated health and enhance their options for seeking medical advice or prevention of disease to reduce the harm caused by chronic diseases [25,26].

In sum, the primary symptoms of cardiovascular disease are not always obvious so they can be easily ignored. Although the prevalence of risk factors in cardiovascular disease is decreasing, there is room for improvement. For example, risk prediction after ACS has been recently boosted with artificial intelligence (AI) [27]. The continuous improvement of health awareness could effectively promote people’s quality of life. The IMPROVE-IT study is just one of the steps in the whole lipid-lowering treatment strategy. A lipid-lowering treatment that can achieve the optimal effect of reducing cardiovascular risk is still needed.

## 5. Conclusions

In the treatment of diabetic patients, higher doses of medication are already used for prognosis treatment. However, the results of this study also found no significant difference in the risk of rehospitalization caused by ACS or stroke between diabetic and non-diabetic patients. This may suggest that the benefits of medication control in diabetes may be reflected after recovery from ACS, but it may also be due to the protective effect of medication for glycemic control in the diabetic patients themselves. For example, the SGLT2 inhibitor, which has cardiac and renal protective effects (Dapagliflozin, Canagliflozin, Empagliflozin), is a commonly used drug for the treatment of diabetes mellitus. However, IMPROVE-IT still provided strong evidence for high intensity statin therapy in secondary prevention [10]. That is to say that the lowest LDL-C level can be provided by high intensity statin and the patient’s lifestyle. However, this study merely discusses the risk of rehospitalization, which is different from previous research on the risk of death.

## Figures and Tables

**Table 1:** Characteristics among inpatients.

	Total	Non-Diabetic	Diabetic	*p*-Value
	*n* (%)	*n* (%)	*n* (%)	
Gender				0.3374
Male	227 (62.36)	131 (64.53)	96 (59.63)	
Female	137 (37.64)	72 (35.47)	65 (40.37)	
Age, years (mean ± SD)	67.49 ± 14.32	68.48 ± 14.56	66.24 ± 13.95	
Age group				0.0160
20–44	26 (7.14)	15 (7.39)	11 (6.83)	
45–64	127 (34.89)	68 (33.50)	59 (36.65)	
65–84	172 (47.25)	89 (43.84)	83 (51.55)	
85+	39 (10.71)	31 (15.27)	8 (4.97)	
Principal diagnosis				0.0064
Unstable angina	34 (9.34)	10 (4.93)	24 (14.91)	
NSTEMI	142 (39.01)	88 (43.35)	54 (33.54)	
STEMI	175 (48.08)	99 (48.77)	76 (47.20)	
Acute coronary syndrome	13 (3.57)	6 (2.96)	7 (4.35)	
Rehospitalization due to ACS or stroke within 1 year				0.8246
YES	37 (10.16)	20 (9.85)	17 (10.56)	
NO	327 (89.84)	183 (90.15)	144 (89.44)	

NSTEMI = non-ST-elevation myocardial infarction; STEMI = ST-elevation myocardial infarction; ACS = acute coronary syndrome.

**Table 2:** Summarized statistics of LDL-C by non-diabetics and diabetics.

	All	Non-Diabetic	Diabetic	*p*-Value ^1^
				0.0883
Mean	93.90	88.24	99.44	
Std	35.32	29.70	39.44	
Q1	67.00	65.00	71.00	
Median	88.00	85.00	90.00	
Q3	110.00	105.00	120.00	

LDL-C = low-density lipoprotein cholesterol. ^1^ Wilcoxon two-sample test.

**Table 3:** Logistic regression analysis result (rehospitalization due to ACS or stroke within 1 year as the response variable).

Variable	Univariate Analysis			Multivariate Analysis		
	OR	95% CI	*p*-Value	OR	95% CI	*p*-Value
Diabetes			0.8246			0.8639
Yes	1.08	0.55–2.14		0.94	0.46–1.93	
No	1.00			1.00		
Gender			0.7404			0.9176
Male	1.13	0.55–2.30		0.96	0.42–2.17	
Female	1.00			1.00		
Age			0.2709			0.2570
20–44	3.36	0.57–19.89		4.06	0.62–26.41	
45–64	2.86	0.63–12.96		3.27	0.65–16.54	
65–84	1.64	0.36–7.53		1.73	0.36–8.18	
85+	1.00			1.00		
Principal diagnosis			0.0970			0.0890
Unstable angina	3.11	0.34–28.16		2.82	0.31–26.10	
NSTEMI	1.63	0.20–13.35		1.72	0.21–14.36	
STEMI	0.88	0.11–7.38		0.80	0.09–6.84	
Acute coronary syndrome	1.00			1.00		

NSTEMI = non-ST-elevation myocardial infarction; STEMI = ST-elevation myocardial infarction.

## Data Availability

Not applicable.
